# Plant Traits as Potential Drivers of Timber Value in the Dipterocarpaceae

**DOI:** 10.1002/ece3.72712

**Published:** 2026-01-05

**Authors:** Nazrin Malik, David Edwards, Robert P. Freckleton

**Affiliations:** ^1^ Ecology and Evolutionary Biology, School of Biosciences University of Sheffield Sheffield UK; ^2^ Department of Forestry Science and Biodiversity, Faculty of Forestry Universiti Putra Malaysia Serdang Selangor Malaysia; ^3^ Department of Plant Sciences and Centre for Global Wood Security University of Cambridge Cambridge UK; ^4^ Conservation Research Institute University of Cambridge Cambridge UK

**Keywords:** Dipterocarpaceae, phylogenetic analysis, plant traits, timber market value, tropical timber, wood density

## Abstract

Southeast Asian tropical forests are vital sources of high‐value timber and non‐timber forest products (NTFPs). This study investigates the relationship between plant traits, wood density, and timber market value within the Dipterocarpaceae family, a critical contributor to the global tropical timber trade and a key structural component of many forests in Southeast Asia. Using a phylogenetic approach, we explored the correlation of morphological and life‐history traits with timber price. Our results show that wood density is significantly associated with higher timber prices, and this relationship is strongly influenced by phylogenetic dependence. We found no evidence linking timber value to the conservation status of dipterocarp species, suggesting that economic exploitation does not necessarily correlate with species endangerment. These findings emphasize the importance of understanding the evolutionary patterns driving economically valuable traits in timber species, which can guide sustainable forest management and conservation strategies in Southeast Asia.

## Graphical abstract

This study examines how plant traits and wood density influence timber market value in Southeast Asia's Dipterocarpaceae family. Using a phylogenetic approach, it finds that higher wood density is linked to greater timber prices, while conservation status does not correlate with market value. The results highlight evolutionary influences on economically valuable traits, with implications for sustainable forest management.

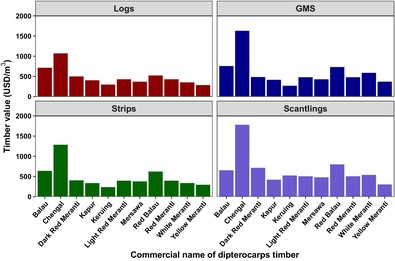

## 1 Introduction

Southeast Asian tropical forests are rich in natural resources with high commercial value (Barreto et al. 1998; de Beer and McDermott 1989; Ghazoul 2016). In particular, this region produces tropical hardwood resources such as timber, veneer, and plywood for a range of wood industries globally (Berry et al. 2010). Historically, countries including Malaysia, Indonesia, and Thailand have dominated the global tropical timber trade by up to 80% (Peluso et al. 1995). The Dipterocarpaceae is one of the plant families that provide significant quantities of timber, plywood, and NTFPs such as resin, dammar, balsam, and essential oils (Ashton 1988; Ghazoul 2016). Dipterocarp timber species are widely distributed in Asian tropical dipterocarp forests and have been of major importance in the global timber trade in the 20th and 21st centuries (Appanah and Turnbull 1998; Sasaki et al. 2016; Roopsind et al. 2018).

Malaysia and Indonesia generate the most dipterocarp timber, especially from Borneo which possesses the highest diversity and abundance of dipterocarps (Maycock et al. 2012). The timber economy has historically contributed a major source of income in these countries. Indonesian timber exports generated about US$6 billion through annual export revenue, of which 60% came from Kalimantan, Borneo. In Malaysia, timber and wood‐based products contributed approximately US$6.8 billion to the national economy in 2010, representing a major component of the country's export revenue (Ghazoul 2016; Pemandu 2010).

Increasing demands on timber supply have caused large areas of lowland dipterocarp forest to be extensively logged in recent decades, leading to concerns about conservation status (Sohngen et al. 1999; Fisher, Edwards, Larsen, et al. 2011; Fisher, Edwards, Giam, and Wilcove 2011). Consequently, a wide range of forest management practices have been adopted in Asian dipterocarp forests to promote sustainable timber and forest resources. Selective logging, reduced impact logging (RIL), and selective management systems (e.g., involving shorter cutting cycles of 30 years and lower diameter limits) have been introduced for forest management in Southeast Asia to assist in managing dipterocarp forests following timber harvesting (Chiew Thang 1987, FAO 2010).

Logging and land‐use conversion can reduce dipterocarp genetic diversity by removing mature reproductive individuals, disrupting pollen flow, and increasing inbreeding (Lee et al. 2000; Obayashi et al. 2002). Reduced genetic variability can influence growth performance and wood characteristics through decreased adaptability and vigor. In this study, “genetic traits” refer to heritable attributes such as chromosome number, ploidy level, and outcrossing rate, which affect evolutionary potential and may indirectly influence timber quality. A study by Palmer et al. (1998) highlighted the importance of timber tree genetics in shaping global timber price value as the genetic traits influence the quality and characteristics of timber, which in turn affects its price in the international market. By assessing the relationship between genetic traits and timber value, it will provide useful information for forest managers to sustain high‐quality timber resources in terms of sustainable forest management and conservation strategies.

Exploitation of Southeast Asian tropical forests mainly results from the high demand for dipterocarps in the timber market because of their high commercial value. High rates of exploitation of dipterocarps in South East Asian forests (Fisher, Edwards, Larsen, et al. 2011; Fisher, Edwards, Giam, and Wilcove 2011; Hawthorne et al. 2011) could be expected to affect dipterocarp conservation status. For instance, conservation assessments by Maycock et al. (2012) projected that approximately 97% of the 33 dipterocarp species assessed in Sabah would qualify as “Threatened” under IUCN Red List criteria. Although this represents only a subset of the > 600 species in the family, it highlights the widespread vulnerability of commercially exploited taxa. However, it is not yet clear whether exploitation driven by high timber value has affected conservation status.

Dipterocarp species differ in their economic value depending on their wood characteristics (Slik 2006; Nock et al. 2009), with higher wood density positively correlated with timber market value due to its influence on mechanical strength, durability, and processing quality (Panshin and Zeeuw 1980; Blakenhorn 2001; Chave et al. 2009). Wood characteristics are the outcome of natural selection in response to the habitat conditions in which tree species evolved, with ecological functional traits that relate to recruitment, survival, growth rate, and shade tolerance particularly important in producing enhanced timber qualities and financial values. Wood density and functional traits are thus evolutionarily interlinked, reflecting adaptive strategies for mechanical support and resource use (King et al. 2005; Chave et al. 2009; Swenson and Enquist 2007), and their association with timber value and commercial desirability arises indirectly (Panshin and Zeeuw 1980; Blakenhorn 2001; Chave et al. 2009). For instance, shade tolerance, maximum diameter at breast height (DBH), and height indicate a species' growth and survival potential and structural investment (King et al. 2005, 2006; Poorter and Bongers 2006; Chave et al. 2009; Niinemets and Valladares 2006; Sterck et al. 2003), while seed weight represents energy allocation during early growth and indirectly affects wood formation and fiber structure (Westoby et al. 1996; Vazquez and Vilas 1998; Moles et al. 2007; Rawat 2022; Longui et al. 2011). An improved understanding of relationships between wood density, life‐history traits, and timber value is essential in sustainable timber resources and global timber value.

Life‐history, morphological, and ecological traits are non‐randomly distributed across species with respect to evolutionary history and phylogeny. Consequently, analyses of traits with respect to phylogeny have provided many fundamental insights (Gamage et al. 2006; Sjöström and Gross 2006; Qian and Zhang 2014). Phylogeny has been used widely in helping to answer questions in ecology and evolutionary biology, patterns in community, and environmental variability in studies across multiple species (Felsenstein 1985; Ackerly 2004; Wiens et al. 2010; Cooper et al. 2011; Liu et al. 2012).

In summary, we expect that the economic value of species will correlate with species' traits, and this will also generate non‐random distributions of value with respect to phylogeny owing to the imprint of evolutionary history on traits. However, to our knowledge, there is no phylogenetic comparative analysis of how species traits covary with economic value. Addition of phylogenetic information could provide a vital way to assess how traits variance influence timber price value as well as promoting sustainable high‐quality timber. For instance, closely related species that share similar traits might also tend to have similar timber price value (Chiew Thang 1987; Tnah et al. 2012). Including phylogeny together with trait data and prices should allow us to build a complete picture of the biotic factors driving timber values.

In this study, we use a phylogeny of the Dipterocarpaceae to assess the relationship between plant traits and timber values for dipterocarp species in Southeast Asia. We have three specific objectives. First, we assess the relationship between dipterocarp wood density and timber price. Second, we evaluate how environmental, morphological, flowering, and genetic traits selected for their potential functional links to wood properties relate to timber market value. Finally, we test whether timber price influences conservation status.

## 2 Materials and Methods

### 2.1 Study Group and Phylogenetic Tree

The Dipterocarpaceae primarily occur in tropical lowland rainforests and contain 17 genera with 695 known species (Christenhusz and Byng 2016). Dipterocarp timber trade is a key economic driver in Southeast Asia, supporting employment, export revenues, and timber‐based manufacturing sectors, while closely linked to sustainable forest management (Sohngen et al. 1999). We used a phylogenetic tree for the Dipterocarpaceae family that we built using the R package “S. PhyloMaker.” We constructed a phylogenetic tree by grafting dipterocarp genera and species included in this study onto a backbone phylogenetic hypothesis (Qian and Jin 2016). We used the PhytoPhylo mega‐phylogeny as the backbone (Qian and Jin 2016), and an updated and expanded version of Zanne et al.'s species‐level phylogeny (Zanne et al. 2014). Zanne et al.'s phylogeny comprises about 30,771 seed plants and was time‐calibrated for all branches using seven gene regions available in GenBank as well as fossil data. Moreover, PhytoPhylo includes all families of extant seed plants (Qian and Zhang 2014) with five times more genera and over 55 times more species than the newest angiosperm supertrees (i.e., R20120829; Qian and Jin 2016).

For genera and species that were not found or missing from the PhytoPhylo mega‐phylogeny, we took three approaches: (1) adding genera or species as polytomies within their families; (2) randomly adding genera or species within their families or genera; and (3) adding genera or species to their families or genera with the same approach used in the online software Phylomatic and BLADJ (branch length adjuster). Using these three approaches, three phylogenies were generated at each level of resolution such as family, genus, and species.

The three approaches for adding missing genera and species to the PhytoPhylo mega‐phylogeny differed in their taxonomic resolution. First, the polytomy approach inserted missing taxa as unresolved nodes within their respective families, preserving phylogenetic uncertainty. Second, the random insertion approach placed missing taxa at random positions within the corresponding genus or family, maintaining overall branch length distribution. Third, the BLADJ‐adjusted approach used estimated divergence times from fossil‐calibrated nodes to assign approximate branch lengths for missing taxa, following the procedure in *Phylomatic* and *BLADJ*.

All three approaches produced highly similar topologies. The final analyses used the BLADJ‐adjusted tree (Scenario 3), as it provided the most resolved branch length information while maintaining phylogenetic consistency across taxa. The other two trees were retained for sensitivity checks (Appendix C2: Data S2), which confirmed that model outcomes were robust to tree topology variation.

### 2.2 Plant Traits and Timber Price Values

Plant trait data for 544 Dipterocarpaceae species were compiled from three main sources: (i) published literature obtained through a comprehensive search using Google Scholar (search term: “Dipterocarpaceae”), (ii) key taxonomic and ecological monographs by Symington (1974) and Ghazoul (2016), and (iii) reputable online plant databases, including the IUCN Red List and PlantUse.net.

In our literature search, we reviewed titles and abstracts from approximately 13,400 Google Scholar results using the keyword “Dipterocarpaceae.” Studies were selected based on the following criteria: (i) inclusion of quantitative or categorical data for at least one of the target plant traits; (ii) focus on wild or natural populations of Dipterocarpaceae rather than experimental or plantation settings; (iii) clear reporting of measurement methods or species‐level means; and (iv) publication in peer‐reviewed journals, monographs, or technical reports. Duplicated records or sources lacking explicit trait data were excluded.

To understand the biological and economic variation among species, we classified plant traits into four functional categories: ecological/environmental, morphological, flowering, and genetic. This classification reflects both ecological function and evolutionary origin. We focused on these traits because they are functionally and phylogenetically linked to wood properties known to determine timber strength, density, and commercial classification.

*Ecological and environmental traits*: (i.e., elevation range, soil type) describe species' habitat preferences and niche specialization, which influence geographic distribution, local abundance, and timber availability across environmental gradients.

*Morphological traits* such as tree height, diameter at breast height (DBH), leaf length, growth rate, and shade tolerance represent life‐history strategies that are related to structural investment, resource acquisition, and survival. These traits directly affect wood formation, density, and commercial yield.

*Flowering traits: For example*, flowering frequency are linked to reproductive phenology and population regeneration. These characteristics shape long‐term timber supply by influencing species' capacity to recover from disturbance and sustain harvestable populations.

*Genetic traits*: these include chromosome number and polyploidy and provide understanding into evolutionary lineage and genetic diversity. Such traits may underlie the key phenotypic variation in growth, wood characteristics, and reproductive success.

Ecological and environmental traits (e.g., elevation range, soil type, geographic distribution) were primarily derived from the monographs of Symington (1974) and Ghazoul (2016), complemented by habitat and distribution data from the IUCN Red List. Morphological traits (e.g., tree height, DBH, leaf length, shade tolerance, growth rate) were obtained from field‐based studies and taxonomic descriptions available in Symington (1974), Ashton (1988), and Nock et al. (2009). Flowering and reproductive traits (e.g., flowering frequency, anthesis timing, fruit size, seed weight, functional wing morphology) were compiled from phenological studies by Appanah and Turnbull (1998), Krisnapillay and Tompsett (1998), and Newman et al. (1998). Genetic traits (e.g., chromosome number, polyploidy, and outcrossing rate) were gathered from molecular phylogenetic and genetic diversity studies (e.g., Gamage et al. 2006; Obayashi et al. 2002; Tnah et al. 2012). Trait values from these sources were cross‐checked among references for consistency, and where multiple estimates existed, mean values were calculated.

For each trait, values were extracted or calculated at the species level. When multiple records existed for a single species, we used the mean of reported values to represent the species trait. Trait data were preferentially taken from adult, mature trees in natural forest conditions, reflecting the developmental stage typically harvested for timber. This approach reduces ontogenetic and environmental variation and ensures comparability among species. When data were available from both field measurements and secondary sources, field‐based or directly measured values were prioritized.

The complete list of traits, definitions, and data sources is provided in Table S1, while their ecological significance and relevance to timber value are summarized in Table S2. Table S1 lists the plant traits of Dipterocarpaceae species used in this study, including trait definitions, measurement units, and class descriptions. Table S2 summarizes these traits by functional category, providing their ecological significance and relevance to timber value to illustrate the functional and evolutionary pathways through which traits may influence wood properties and market price. These trait categories were selected for their biological relevance to forest dynamics, timber quality, and species' responses to exploitation. Their functional and phylogenetic significance makes them strong candidates for predicting timber market value, which is influenced by wood properties, ecological resilience, and species availability. This framework also enabled us to account for phylogenetic non‐independence in our comparative analyses.

For timber price value, we collected data from the International Tropical Timber Organization (ITTO) website (https://www.itto.int) and Malaysian Timber Industry Board (MTIB) timber price database (http://www.mtib.gov.my). Timber price data were collected from the International Tropical Timber Organization (ITTO) and Malaysian Timber Industry Board (MTIB) databases for the period 2010–2020. Preliminary checks across multiple years indicated consistent relative rankings among species, suggesting that interspecific price differences remained stable over time despite market fluctuations. In this study, we used logs and sawn timber price value (i.e., GMS, Scantlings, and Strips) to assess the relationship with wood density. According to ITTO and MTIB, timber logs are the unprocessed raw timber tree trunks without branches with a minimum diameter of 15.24 cm and minimum length of 1.2 m. General market specification (GMS) sawn timber is a wood product made from logs that have been sawed or second sawed with a minimum of two sides of surfaced logs that have been sawed according to general timber market specifications. Scantlings are timber products sawn from logs to standardized dimensions, typically 2.5–10 cm in width and 46–183 cm or longer in length. Strips are sawn timber of smaller size, typically 2.5 × 2.5 cm, used where narrow or thin pieces are required. In this study, we used United States Dollar (USD) as the unit of currency throughout.

### 2.3 Phylogenetic Analysis

We used Pagel's *λ* to test for phylogenetic dependence based on a Brownian model of trait variation (Pagel 1999; Freckleton et al. 2002). This parameter varies between zero and one: *λ* = 0 suggests that there is no phylogenetic dependence, and *λ* = 1 suggests perfect phylogenetic dependence, as predicted by a Brownian model.

To achieve our first objective, linear models were fitted by using species' timber values as the response variable, while species' elevation, distribution, habitat soil type, morphological traits, genetic traits, seed traits, and wood type were entered as predictors. To address the second objective, we used wood density as the predictor variable, and timber and sawn timber price values as response variables in the models. For the third objective, we constructed linear models to test the relationship between morphological and life‐history strategies traits as predictors, with wood density as the response variable. To test how timber price value might affect conservation status, we fitted linear models with conservation status using IUCN Red List classifications, population trend, habitat destruction, and% habitat decline as responses, while timber value was entered as the predictor variable.

Linear models were fitted using the pgls function in the R package (Orme et al. 2013). The *λ* statistic was used to control for phylogenetic non‐independence in the residuals of linear models (e.g., following Freckleton et al. 2002). We used these models for testing relationships between plant traits, wood density, and conservation status with timber value, and for building models for association between morphological traits and wood density. We estimated *λ* values in models of each trait correlated with timber price by using the pgls function from the R package caper (Orme et al. 2013).

## 3 Results

### 3.1 Relationship Between Plant Traits and Global Timber Price

We found no significant relationship between elevation, geographic distribution, habitat soil type, height, or DBH with global timber price value (Table 1). The growth rate of dipterocarp species showed a weak significant association with timber price value, with no phylogenetic dependence (*p* ns for *λ* = 0; Table 1). Dipterocarp flowering frequency revealed a weak significant association with timber price value, with strong phylogenetic dependence (*λ* = 0.839, *p* < 0.001 for *λ* = 0; Table 1).

**TABLE 1:** Model coefficients, *F* and *λ* values for phylogenetic linear models testing the relationship between timber price value (logs/ton) and plant traits.

Trait category	Trait	Units	Min–Max–Median	*n*	Model coefficient (Estimate ± SE)	*F*/*p* value	*λ*	*p* (*λ* = 0)	*p* (*λ* = 1)
Ecological/Environmental traits	Lower elevation limit	m	0–1400–0	320	0.02 ± 0.04	0.151^ns^	0.852	***	***
	Upper elevation limit	m	0–1800–650	320	1.12e‐03 ± 2.04e‐02	0.003^ns^	0.852	***	***
	Geographic distribution (Widespread/Endemic)	binary	0–1–1	226	−19.63 ± 13.22	2.206^ns^	0.857	***	***
	Extent of occurrence	km^2^	0.29–8,872,598–20,001	93	−8.34e‐06 ± 7.23e‐06	1.333^ns^	0.778	***	***
	Soil type (Clay)	0/1	0–1–1	173	−23.88 ± 14.06	2.883^ns^	0.880	***	***
	Soil type (Sandy)	0/1	0–1–1	173	7.73 ± 13.70	0.318^ns^	0.875	***	***
	Soil type (Loam)	0/1	0–1–0	173	−36.26 ± 37.04	0.959^ns^	0.871	***	***
	Soil type (Limestone)	0/1	0–1–0	173	−28.58 ± 33.67	0.720^ns^	0.873	***	***
Morphological traits	Height	m	5–82–40	194	0.48 ± 0.68	0.505^ns^	0.827	***	***
	Diameter at breast height	cm	5–310–110	195	0.04 ± 0.16	0.070^ns^	0.830	***	***
	Growth rate	cm/year	0.2–70–1.25	16	−51.38 ± 22.48	5.226*	0.000	ns	*
	Shade tolerance	ordinal (0–2)	0–2–1	101	−12.49 ± 10.59	1.391^ns^	0.878	***	***
	Leaf length	cm	2.5–45–12	204	−1.24 ± 1.21	1.049^ns^	0.842	***	***
	Seed weight	seeds/kg	30–45,000–930	39	0.02 ± 0.01	5.424*	0.982	***	**
	Seed wing morphology: wingless	0/1	0–1–0	227	703.67 ± 68.40	105.830***	0.892	***	***
	Functional wing = 2	0/1	0–1–0	227	−25.32 ± 48.34	0.274^ns^	0.854	***	***
	Functional wing = 5	0/1	0–1–1	227	−74.18 ± 31.95	5.392*	0.868	***	***
	Wing length	mm	1.8–90–8.5	69	−0.35 ± 0.65	0.287^ns^	0.973	***	***
	Wood type (timber category)	Ordinal (0–2)	0–2–1	227	117.92 ± 9.43	156.330***	0.932	***	***
Flowering traits	Flowering frequency	0/1	0–1–0	227	−59.42 ± 23.84	6.212*	0.839	***	***
Genetic traits	Chromosome number (*x* = 7)	0/1	0–1–1	227	127.38 ± 32.15	15.702***	0.833	***	***
	Chromosome number (*x* = 10)	0/1	0–1–0	227	−152.99 ± 36.94	17.152***	0.833	***	***
	Chromosome number (*x* = 11)	0/1	0–1–1	227	−127.38 ± 32.15	15.702***	0.833	***	***
	Polyploidy	0/1	0–1–1	227	−180.78 ± 40.61	19.814***	0.887	***	***

*Note:* Binary traits (0/1) indicate the presence (1) or absence (0) of a characteristic or category, e.g., soil type, flowering frequency, or chromosome number. Ordinal traits represent categorical variables with a natural order but not necessarily equal intervals, for example, shade tolerance (0–2) or timber category (0–2). *p*‐values: ns = not significant; *p < 0.05; **p < 0.01; ***p < 0.001. Units and value ranges (Min–Max–Median) for each trait are shown. *n* represents the number of dipterocarp species analyzed for each model.

In terms of genetic traits in dipterocarp species, there were significant associations between all chromosome number and timber price value, with strong phylogenetic dependence (*λ* = 0.833, all *p* < 0.001 for *λ* = 0; Table 1). Polyploidy also showed a statistically significant relationship with timber price (*λ* = 0.887, *p* < 0.001 for *λ* = 0; Table 1).

**FIGURE 1:**
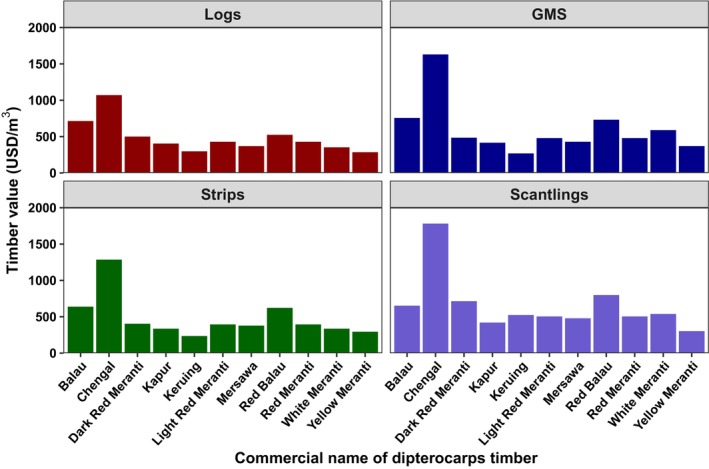
Timber price value (USD/m^3^) for dipterocarp commercial names (Heavy hardwood = Chengal, Balau, and Red Balau; Medium hardwood = Keruing, and Kapur; Light Hardwood = Dark Red Meranti, Light Red Meranti, Red Meranti, Yellow Meranti, and White Meranti).

Of the seed traits, seed weight showed a weakly significant relationship with timber price and strong phylogenetic dependence (*λ* = 0.982, *p* < 0.001 for *λ* = 0; Table 1). From seed's functional wing traits, wingless seed showed a strong significant association with timber price and strong phylogenetic dependence (*λ* = 0.892, *p* < 0.001 for *λ* = 0; Table 1). Wood type showed a strong significant relationship with timber price and strong phylogenetic dependence (*λ* = 0.932, *p* < 0.001 for *λ* = 0; Table 1; Figure 1).

### 3.2 Wood Density and Timber Price Value

**FIGURE 2:**
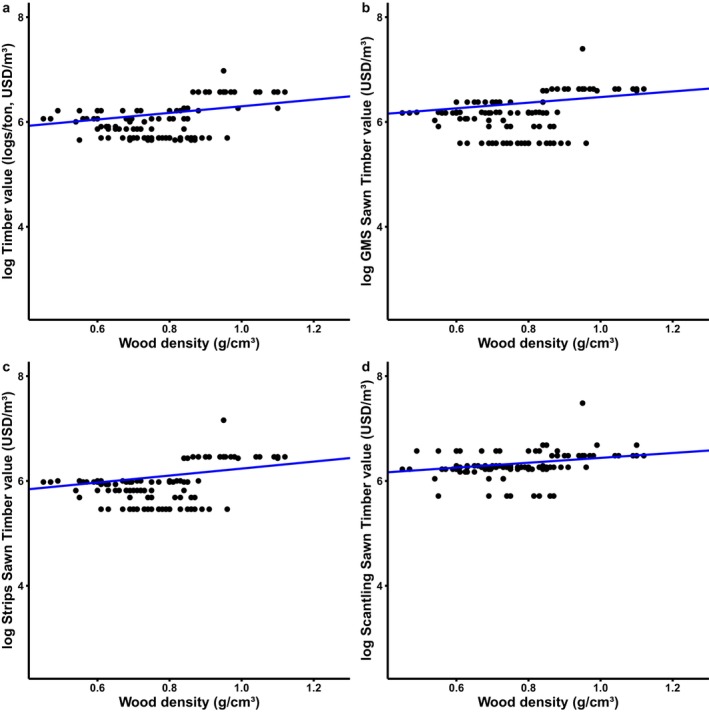
Relationship between wood density (g/cm^3^) and timber price value (USD/m^3^) across dipterocarp species. Each point represents one species. Timber price values were log‐transformed prior to analysis.

Wood density showed a statistically significant association with timber price for GMS, and strong phylogenetic dependence (*λ* = 0.973, *p* < 0.001 for *λ* = 0; Table 2; Figure 2a). Timber logs, strips, and scantlings of sawn timber were significantly correlated with wood density and exhibited phylogenetic dependence, with *λ* values ranging from 0.810 to 0.943. (all *p* < 0.001 for *λ* = 0; Table 2, Figure 2b–d).

**TABLE 2:** Model coefficient, *F* and *λ* values for phylogenetic linear model testing the relationship of timber price value and wood densities (values are log‐transformed).

	*n*	Model coefficient	*F* ^*p*^ value	*λ*	*p* (*λ* = 0)	*p* (*λ* = 1)
		(Estimate ± SE)				
(a) Timber (logs/ton)						
Wood density	136	0.630 ± 0.11	32.778***	0.900	***	***
(b) GMS (sawn timber/m^3^)						
Wood density	136	0.536 ± 0.10	31.421***	0.973	***	***
(c) Strips (sawn timber/m^3^)						
Wood density	136	0.667 ± 0.11	36.222***	0.943	***	***
(d) Scantlings (sawn timber/m^3^)						
Wood density	136	0.465 ± 0.12	15.744***	0.810	**	***

*Note:* *p < 0.05; **p < 0.01; ***p < 0.001.

Abbreviation: ns, not significant.

### 3.3 Relationship Between Wood Density and Morphological and Life‐History Traits

We found no significant relationship between wood densities with height, diameter, growth rate, and survival. However, there was phylogenetic dependence observed in the height and diameter models with *λ* value 0.419 and 0.451, respectively (all *p* < 0.001 for *λ* = 0; Table 3). Shade tolerance traits were significantly associated with wood density, with moderate phylogenetic dependence (*λ* = 0.574, *p* < 0.001 for *λ* = 0; Table 3). Strong associations were observed in the phylogenetic model between wood density and wood type, but with weak phylogenetic dependence (*λ* = 0.412, *p* < 0.001 for *λ* = 0; Table 3).

**TABLE 3:** Model coefficient, *F* and *λ* values for phylogenetic linear models of wood density on morphological traits and life‐history traits.

	*n*	Model coefficient	*F* ^*p*^ value	*λ*	*p* (*λ* = 0)	*p* (*λ* = 1)
		(Estimate ± SE)				
(a) Wood density						
Height	221	−0.0010 ± 0.0007	3.852^ns^	0.419	***	***
Diameter at breast height	224	−0.0003 ± 0.0001	3.126^ns^	0.451	***	***
Growth rate	23	−0.0014 ± 0.0026	0.278^ns^	0.000	ns	***
Survival	40	−0.0039 ± 0.0021	3.324^ns^	0.000	ns	***
Shade tolerance	124	−0.0590 ± 0.0205	8.087**	0.574	***	***
Wood type	236	0.0970 ± 0.0120	70.390***	0.412	**	***

*Note:* *p < 0.05; **p < 0.01; ***p < 0.001.

Abbreviation: ns, not significant.

### 3.4 Timber Price and Conservation Status

We found no significant associations between timber price and conservation status of dipterocarp plant species, as well as population trend, habitat destruction, and percentage of habitat decline. Nevertheless, phylogenetic dependence was observed in all models, with *λ* value ranging from 0.854 to 0.867 (all *p* < 0.001 for *λ* = 0; Table 4).

**TABLE 4:** Model coefficient, *F* and *λ* values for phylogenetic linear models of timber price value on conservation status.

	*n*	Model coefficient	*F* ^*p*^ value	*λ*	*p* (*λ* = 0)	*p* (*λ* = 1)
		(Estimate ± SE)				
(a) Timber (logs/ton)						
Red List status	169	−1.313 ± 7.996	0.027^ns^	0.867	***	***
Population trend	227	2.908 ± 16.421	0.031^ns^	0.854	***	***
Habitat destruction	227	−2.023 ± 14.968	0.018^ns^	0.854	***	***
Percentage of habitat decline	227	5.318 ± 5.380	0.977^ns^	0.856	***	***

*Note:* *p < 0.05; **p < 0.01; ***p < 0.001.

Abbreviation: ns, not significant.

## 4 Discussion

We found a significant relationship between plant traits and timber price value. Wood densities are highly correlated with timber price value, and there are also associations with several genetic traits. We do not find any evidence that timber price value affects the conservation status of dipterocarps. This is likely because all dipterocarps are harvested in areas where logging occurs. In other words, the harvesting is not specifically selective for dipterocarp species but targets all species present in the area given. Thus, our overall finding is that biotic factors act as vital components in driving timber price value.

### 4.1 Plant Traits and Global Timber Price Value

We found no relationship between environmental variables such as elevational gradient, geographic distribution, and habitat soil types with timber price value. This suggests that dipterocarp species' environmental adaptations do not affect economic value. Vincent et al. (2002) examined the pricing of tropical timber and found that market dynamics and species‐specific characteristics, such as wood quality and rarity, often outweigh ecological or environmental factors in determining timber prices.

Flower size traits showed a significant relationship with timber price value for dipterocarp species. This result could be due to flower characteristics exhibited by high‐value dipterocarp species. For example, small‐flowered species such as *Neobalanocarpus* hemii (Chengal) have the highest timber price value in the Malaysia timber market (Tnah et al. 2012). We found weak evidence of a correlation of flowering frequency with timber price. Dipterocarps species like *Shorea* spp. are usually involved in general flowering events, but there are many species in Sumatra and Borneo from *Dipterocarpus* (Keruing) and *Dryobalanops* (Kapur), including *Neobalanocarpus hemii* in Peninsular Malaysia that flower annually or biannually (Krisnapillay and Tompsett 1998).

We found little evidence of dipterocarp growth rate traits impacting timber price value. Fast‐growing dipterocarp species (i.e., *Parashorea* spp., *Anisoptera* spp. and some *Shorea* spp.) have usually been logged frequently due to increasing demand in the timber market (Yeong et al. 2016). Unfortunately, we do not have sufficient power in our analysis to make strong conclusions about the influence of growth rates on timber price because we have growth rate data on only 16 of 228 species. Although morphological traits such as height and diameter, survival, shade tolerance traits are major factors in influencing plant growth for the next cutting cycle for logging (Ådjers et al. 1995; Kuusipalo et al. 1996), in our analysis we found no evidence of morphological traits, survival, and shade tolerance traits on timber price value.

Although some morphological and reproductive traits did not directly predict timber price, their ecological links to wood properties remain important. For instance, larger‐seeded or shade‐tolerant species typically invest in denser, more durable wood, whereas fast‐growing species tend to produce lighter wood with lower market value. These indirect relationships emphasize that trait influences on timber economics often act through their association with wood density rather than independently.

### 4.2 Wood Densities Impact on Timber Price Value

In our study, we found a significant relationship between wood density for timber logs and sawn timbers with timber price value with strong phylogenetic dependence. This result is linked to the result that higher wood density has a higher timber price value. For example, Chengal (*Neobalanocarpus*) and Shorea species, such as Balau (*Shorea*) and Red Balau (*Rubroshorea*), have wood densities ranging from 800 to 1160 kg/m^3^ and are classified as heavy hardwoods by foresters due to their high timber value in the market (Lopez 1981; Lopez 1983). *Neobalanocarpus hemii* is a heavy hardwood species that occurs in Peninsular Malaysia; it is highly valued (see Figure 2) and well‐known timber where Malaysia is the only exporter of *Neobalanocarpus hemii* sawn timbers (Lopez 1983).

Most Asian dipterocarps are classified by foresters based on their wood density, wood type and commercial value and exclude species with little or no commercial value (Symington 1941; Ashton 1985). Furthermore, from engineering perspectives, strong and durable wood exhibits a high price of timber value. Wood density is correlated to the mechanical properties of wood in that as density increases, the strength of wood increases (Blakenhorn 2001). Our findings highlight that wood density is an indicator in determining timber price value.

### 4.3 Wood Density on Morphological Traits and Life‐History Strategies Traits

King et al. (2005) revealed that tree growth is expected to be associated with wood density since the volume of wood produced with a given unit of biomass is inversely proportional to its density. However, we found no evidence when modeling wood density with morphological traits such as height, DBH, survival, and growth rate. This might be due to a lack of growth rate data in our analysis.

Verburg et al. (2003) noted that wood density is closely associated with the life‐history strategies of tree species. For example, recent studies have shown that there is a relationship between wood density and the successional stage a species occupies (Chen et al. 2017; Charles et al. 2018). In our study, we found significant evidence of a correlation of shade tolerance traits with wood density. Our finding supported the suggestion that light‐demanding species have low wood density because it promotes rapid height development in high light conditions by producing low‐density wood (King et al. 2006). Meanwhile, several studies found that shade‐tolerant tree species tend to grow slowly and invest in dense, strong, and damage‐resistant wood that in turn lowers their mortality rates (Putz et al. 1983; Muller‐Landau 2004; van Gelder et al. 2006).

### 4.4 Timber Value, Conservation Status, and Population Trends

In terms of conservation status, we found no evidence that there is an association between conservation status, population trend, and habitat destruction of timber price value. This may be attributed to the fact that timber prices are largely determined by wood type and density, rather than being specific to individual species. Thus, common species will suffice equally as well as rare ones if the timber characteristics fit the desired usage. Very intensive logging, as often happens in the region, as well as conversion to other land uses and agricultural expansion (oil palm and rubber plantations), are more likely to affect the conservation status of dipterocarps but not the value of timbers (Palmer 2001; Jomo et al. 2004; Forrest et al. 2015; Ghazoul 2016). One possible explanation for the weak relationship between IUCN conservation status and timber value is that both common and rare species can possess high‐value wood depending on their density and mechanical properties (Chave et al. 2009; Blakenhorn 2001). Consequently, economic importance does not necessarily align with extinction risk, which is more strongly driven by habitat loss and exploitation intensity than by intrinsic wood value (Asner et al. 2009; Edwards et al. 2014; Laurance et al. 2014).

Despite the strong relationships observed between plant traits and timber value, several limitations should be acknowledged. Timber prices are not static and may fluctuate across regions and time periods due to market demand, logging intensity, trade restrictions, and policy interventions. These socioeconomic and temporal dynamics may influence the strength or direction of the relationships identified here, potentially limiting the generality of our findings beyond the current market context. Another limitation lies in the phylogenetic resolution of the dataset. Although the BLADJ‐adjusted tree provided the most resolved topology available, some species remained in polytomies, particularly at the species level. This unresolved structure may obscure finer‐scale phylogenetic patterns and lead to stronger signals being detected at genus or subgenus levels, where wood type and density tend to be conserved. Future work using more complete molecular phylogenies and broader temporal price data could refine these associations and improve the predictive capacity of trait‐based economic models.

## 5 Conclusion

Our study found that plant traits such as wood density and wood type are most strongly associated with timber price value, likely because these offer more durable wood. Phylogenetic comparative analysis of how species traits vary with economic value is crucial for understanding the evolutionary patterns that have shaped economically important traits, such as wood density, timber price, yield growth rate, or resilience. This study helps in identifying whether these traits are phylogenetically conserved or have evolved independently across different lineages, which can inform sustainable resource management and conservation strategies. By understanding the evolutionary history and relationships of dipterocarp species, we can better predict which species or lineages might possess valuable traits, guiding efforts in forestry and biodiversity conservation to maximize economic benefits while minimizing ecological impact.

## Author Contributions

**Nazrin Malik:** conceptualization (lead), data curation (lead), formal analysis (lead), investigation (lead), methodology (lead), project administration (equal), validation (equal), visualization (equal), writing – original draft (equal), writing – review and editing (equal). **David Edwards:** conceptualization (equal), formal analysis (equal), project administration (equal), supervision (equal), validation (equal), writing – review and editing (equal). **Robert P. Freckleton:** conceptualization (equal), data curation (equal), formal analysis (equal), investigation (equal), methodology (equal), project administration (equal), supervision (lead), validation (lead), visualization (equal), writing – review and editing (equal).

## Funding

The authors have nothing to report.

## Conflicts of Interest

The authors declare no conflicts of interest.

## Supporting information


**Data S1:** ece372712‐sup‐0001‐DataS1.7z.

**Data S2:** ece372712‐sup‐0002‐DataS2.docx.

## Data Availability

The data that support the findings of this study have been provided in the Data S1.
