# Supplementary material for: Plant Traits as Potential Drivers of Timber Value in the Dipterocarpaceae
Source: Ecol Evol. 2026 Jan 5;16(1):e72712. doi: 10.1002/ece3.72712 (PMC12771645; doi:10.1002/ece3.72712)
Supplement: Supplementary file 2 — Data S2: ece372712‐sup‐0002‐DataS2.docx. [file ECE3-16-e72712-s001.docx]

**Table S1**: List of plant traits of dipterocarp species that have been used in this study

| **Traits** | **Definition** | **Units** | **Description of classes** |
| --- | --- | --- | --- |
| **Lower elevation limit** | Low distance above sea level of species occurrence | m | Quantitative value |
| **Upper elevation limit** | High distance above sea level of species occurrence | m | Quantitative value |
| **Endemism** | Species that being unique to specific location |  | Qualitative (Widespread = 0, Endemic= 1) |
| **Estimated Extent of Occurrence** | “area contained within the shortest continuous imaginary boundary which can be drawn to encompass all the known, inferred or projected sites of present occurrence of a taxon, excluding cases of vagrancy”, (IUCN 2001) | km^2^ | Quantitative value |
| **Estimated Area of Occupancy** | “area within its 'extent of occurrence' which is occupied by a taxon, excluding cases of vagrancy”, (IUCN 2001) | km^2^ | Quantitative value |
| **Habitat Soil type** | Soil type that inhabited by plant (Voroney, 2007) |  | Qualitative (Soil type= Clay, Sandy, Loam, and Limestone  Inhabiting? Yes= 1, No=0) |
| **Height** | Distance from ground level to the level top of the tree | m | Quantitative value |
| **Diameter at breast height (DBH)** | Measurement of tree stem at the height of 1.30 m | cm | Quantitative value |
| **Growth rate** | Girth increment per year | cm/per year | Quantitative value |
| **Shade tolerance** | Ability to tolerate low light level |  | Qualitative (Shade tolerant= 0, Intermediate= 1, Light demander= 2) |
| **Leaf length** | Length of the leaf in vascular plants from lamina tip to the petioles along lamina midrib (Cho *et al.*, 2007) | cm | Quantitative value |
| **Flower size** | Diameter of flower | mm | Qualitative (Small(<10mm) = 0, Medium(10—20mm) = 1, Large(>20mm) = 2) |
| **Flower reward** | Secretion or structure of the labellum that can be consumed or gathered by pollinators (Singer & Koehler 2004) |  | Qualitative (Type= Nectar, Pollen and Corolla, Produced? Yes= 1, No= 0) |
| **Survival** | Tree mortality | % | Quantitative value |
| **Flowering frequency** | Regularity of flowering in vascular plant |  | Qualitative (General= 0, Regular= 1) |
| **Anthesis (Day)** | Flowering period of plant (0600-1800) |  | Qualitative (Yes= 1, No= 0) |
| **Anthesis (Night)** | Flowering period of plant (1800-0600) |  | Qualitative (Yes=1, No=0) |
| **Chromosome number** | Number of DNA molecule that carry genetic information of plant (Battaglia, 1955) |  | Qualitative (Chromosome no x=7,10,11 and Polyploidy,  Yes=1, No=0) |
| **Outcrossing rate** | Rates of crossing between different breeds | % | Quantitative value |
| **Fruit length** | Length of nut | mm | Quantitative value |
| **Fruit width** | Width of nut | mm | Quantitative value |
| **Seed weight** | Seed mass | seed per kilo | Quantitative value |
| **Functional wing** | Wings that involved in seed dispersal |  | Qualitative (Wing no= 0,2,3 and 5,  Has? Yes= 1, No=0) |
| **Functional wing length** | Measurement of length of wings involved in dispersal | mm | Quantitative value |
| **Wing loading** | Fruit mass divided by wing surface area(Green, 1980) |  | Quantitative value |
| **Wood type** | Hardwood type classification |  | Qualitative (Light Hardwood=0, Medium Hardwood= 1, Heavy Hardwood= 2) |
| **Wood densities** | “Measurement of the ratio of oven-dry mass of wood divided mass of water displaced by its green volume “ (Chave, no date) | g/cm^3^ | Quantitative value |
| **Red List status** | Species conservation status through criteria such as population size, rate of decline and geographic distribution as listed in IUCN Red List Categories (IUCN 2017) |  | Qualitative (Data Deficient= 0, Least Concern= 1, Near Threatened= 2, Vulnerable= 3, Endangered=4, Critically Endangered=5, Extinct in The Wild= 6) |

**Table S2**: Plant traits of Dipterocarpaceae species used in this study, grouped by functional category. Ecological significance and relevance to timber value are provided to illustrate functional and evolutionary pathways through which traits may influence wood

| **Trait** | **Category** | **Ecological significance / relevance to timber value** |
| --- | --- | --- |
| **Lower elevation limit** | Ecological | Reflects environmental tolerance; species from lowland forests often produce denser, slower-grown wood valued in construction. |
| **Upper elevation limit** | Ecological | High-elevation species typically experience slower growth and may form denser wood with higher mechanical strength. |
| **Endemism** | Ecological | Endemic species may have restricted supply, influencing rarity and market value. |
| **Estimated Extent of Occurrence (EOO)** | Ecological | Indicates distribution breadth; widespread species provide consistent timber supply, affecting price stability. |
| **Estimated Area of Occupancy (AOO)** | Ecological | Correlates with population abundance and harvest potential; smaller AOO may increase rarity value. |
| **Habitat soil type** | Ecological | Soil fertility influences growth rate and wood formation; nutrient-poor soils often yield denser wood. |
| **Height** | Morphological | Determines stem form and total yield; taller trees yield longer, higher-quality logs. |
| **Diameter at breast height (DBH)** | Morphological | Indicates growth potential and yield; affects harvestable volume and log quality |
| **Growth rate** | Life-history | Balances productivity and wood density; slower growth usually produces denser, higher-value wood. |
| **Shade tolerance** | Life-history | Correlates with wood density; shade-tolerant species invest in stronger, more durable wood. |
| **Leaf length** | Morphological | Indicates light-capture strategy; indirectly linked to growth and carbon allocation affecting wood formation. |
| **Flower size** | Reproductive | Related to reproductive effort and pollination strategy; may co-vary with life-history pace and growth allocation. |
| **Flower reward** | Reproductive | Reflects pollination ecology; indirectly connected to resource allocation between reproduction and growth. |
| **Survival** | Life-history | High survival often associated with conservative growth strategies and denser wood. |
| **Flowering frequency** | Reproductive | Affects regeneration potential and long-term timber supply sustainability. |
| **Anthesis (Day)** | Reproductive | Pollination timing linked to pollinator assemblages; influences reproductive success and population persistence. |
| **Anthesis (Night)** | Reproductive | As above, nocturnal anthesis may reflect specific ecological strategies affecting reproduction rates. |
| **Chromosome number** | Genetic | Indicates evolutionary lineage and genome size; linked to growth and cell structure influencing wood characteristics. |
| **Outcrossing rate** | Genetic | Genetic diversity promotes vigor and growth stability, affecting timber quality and productivity. |
| **Fruit length** | Reproductive | Represents dispersal mechanism and resource allocation; indirectly tied to growth patterns. |
| **Fruit width** | Reproductive | As above, influences seed investment strategy. |
| **Seed weight** | Reproductive | Heavier seeds yield robust seedlings and slower but denser wood formation. |
| **Functional wing** | Reproductive | Determines seed dispersal distance; indirectly linked to colonization potential and distribution. |
| **Functional wing length** | Reproductive | As above, longer wings enhance dispersal and habitat occupancy. |
| **Wing loading** | Reproductive | Ratio of seed mass to wing area; affects dispersal and establishment, influencing population structure and timber availability. |
| **Wood type** | Morphological | Defines anatomical class (light, medium, heavy hardwood); major determinant of timber strength and price. |
| **Wood density** | Morphological | Determines timber strength, durability, and market class (heavy vs light hardwood) |
| **Red List status** | Conservation | Indicates extinction risk; rare or threatened species may command limited legal trade or conservation-based premium. |

**Appendix C1**: Dipterocarp species list used in the study

| *Anisoptera brunnea* | *Dipterocarpus coriaceus* |
| --- | --- |
| *Anisoptera costata* | *Dipterocarpus cornutus* |
| *Anisoptera curtisii* | *Dipterocarpus costatus* |
| *Anisoptera grossivenia* | *Dipterocarpus costulatus* |
| *Anisoptera laevis* | *Dipterocarpus crinitus* |
| *Anisoptera marginata* | *Dipterocarpus cuspidatus* |
| *Anisoptera megistocarpa* | *Dipterocarpus dyeri* |
| *Anisoptera reticulata* | *Dipterocarpus elongatus* |
| *Anisoptera scaphula* | *Dipterocarpus eurhynchus* |
| *Anisoptera thurifera* | *Dipterocarpus fagineus* |
| *Cotylelobium burckii* | *Dipterocarpus fusiformis* |
| *Cotylelobium lanceolatum* | *Dipterocarpus geniculatus* |
| *Cotylelobium lewisianum* | *Dipterocarpus glabrigemmatus* |
| *Cotylelobium melanoxylon* | *Dipterocarpus glandulosus* |
| *Cotylelobium scabriusculum* | *Dipterocarpus globosus* |
| *Dipterocarpus acutangulus* | *Dipterocarpus gonopterus* |
| *Dipterocarpus alatus* | *Dipterocarpus gracilis* |
| *Dipterocarpus applanatus* | *Dipterocarpus grandiflorus* |
| *Dipterocarpus baudii* | *Dipterocarpus hasseltii* |
| *Dipterocarpus borneensis* | *Dipterocarpus hispidus* |
| *Dipterocarpus bourdillonii* | *Dipterocarpus humeratus* |
| *Dipterocarpus caudatus* | *Dipterocarpus indicus* |
| *Dipterocarpus caudiferus* | *Dipterocarpus insignis* |
| *Dipterocarpus chartaceus* | *Dipterocarpus intricatus* |
| *Dipterocarpus cinereus* | *Dipterocarpus kerrii* |
| *Dipterocarpus concavus* | *Dipterocarpus kunstleri* |
| *Dipterocarpus confertus* | *Dipterocarpus lamellatus* |
| *Dipterocarpus conformis* | *Dipterocarpus littoralis* |
| *Dipterocarpus lowii* | *Dryobalanops fusca* |
| *Dipterocarpus mannii* | *Dryobalanops keithii* |
| *Dipterocarpus megacarpus* | *Dryobalanops lanceolata* |
| *Dipterocarpus mundus* | *Dryobalanops oblongifolia* |
| *Dipterocarpus nudus* | *Dryobalanops rappa* |
| *Dipterocarpus oblongifolius* | *Dryobalanops sumatrensis* |
| *Dipterocarpus obtusifolius* | *Hopea acuminata* |
| *Dipterocarpus ochraceus* | *Hopea aequalis* |
| *Dipterocarpus orbicularis* | *Hopea altocollina* |
| *Dipterocarpus pachyphyllus* | *Hopea andersonii* |
| *Dipterocarpus palembanicus* | *Hopea apiculata* |
| *Dipterocarpus perakensis* | *Hopea aptera* |
| *Dipterocarpus pseudocornutus* | *Hopea auriculata* |
| *Dipterocarpus retusus* | *Hopea bancana* |
| *Dipterocarpus rigidus* | *Hopea basilanica* |
| *Dipterocarpus rotundifolius* | *Hopea beccariana* |
| *Dipterocarpus sarawakensis* | *Hopea bilitonensis* |
| *Dipterocarpus semivestitus* | *Hopea brachyptera* |
| *Dipterocarpus stellatus* | *Hopea bracteata* |
| *Dipterocarpus sublamellatus* | *Hopea brevipetiolaris* |
| *Dipterocarpus tempehes* | *Hopea bullatifolia* |
| *Dipterocarpus tuberculatus* | *Hopea cagayanensis* |
| *Dipterocarpus turbinatus* | *Hopea canarensis* |
| *Dipterocarpus validus* | *Hopea celebica* |
| *Dipterocarpus verrucosus* | *Hopea celtidifolia* |
| *Dipterocarpus zeylanicus* | *Hopea centipeda* |
| *Dryobalanops aromatica* | *Hopea cernua* |
| *Dryobalanops beccarii* | *Hopea chinensis* |
| *Hopea cordata* | *Hopea jucunda* |
| *Hopea cordifolia* | *Hopea kerangasensis* |
| *Hopea coriacea* | *Hopea kitulgallensis* |
| *Hopea dasyrrhachis* | *Hopea latifolia* |
| *Hopea depressinerva* | *Hopea longirostrata* |
| *Hopea discolor* | *Hopea malibato* |
| *Hopea dryobalanoides* | *Hopea megacarpa* |
| *Hopea dyeri* | *Hopea mengarawan* |
| *Hopea enicosanthoides* | *Hopea mesuoides* |
| *Hopea erosa* | *Hopea micrantha* |
| *Hopea exalata* | *Hopea mindanensis* |
| *Hopea ferrea* | *Hopea modesta* |
| *Hopea ferruginea* | *Hopea mollissima* |
| *Hopea fluvialis* | *Hopea montana* |
| *Hopea forbesii* | *Hopea myrtifolia* |
| *Hopea foxworthyi* | *Hopea nervosa* |
| *Hopea glabra* | *Hopea nigra* |
| *Hopea glabrifolia* | *Hopea nodosa* |
| *Hopea glaucescens* | *Hopea novoguineensis* |
| *Hopea gregaria* | *Hopea nutans* |
| *Hopea griffithii* | *Hopea oblongifolia* |
| *Hopea hainanensis* | *Hopea obscurinerva* |
| *Hopea helferi* | *Hopea odorata* |
| *Hopea hongayanensis* | *Hopea ovoidea* |
| *Hopea inexpectata* | *Hopea pachycarpa* |
| *Hopea iriana* | *Hopea papuana* |
| *Hopea jacobi* | *Hopea parviflora* |
| *Hopea johorensis* | *Hopea parvifolia* |
| *Hopea paucinervis* | *Hopea treubii* |
| *Hopea pedicellata* | *Hopea ultima* |
| *Hopea pentanervia* | *Hopea utilis* |
| *Hopea philippinensis* | *Hopea vacciniifolia* |
| *Hopea pierrei* | *Hopea vesquei* |
| *Hopea plagata* | *Hopea vietnamensis* |
| *Hopea polyalthioides* | *Hopea wyatt-smithii* |
| *Hopea ponga* | *Marquesia acuminata* |
| *Hopea pterygota* | *Marquesia excelsa* |
| *Hopea pubescens* | *Marquesia macroura* |
| *Hopea quisumbingiana* | *Monotes adenophyllus* |
| *Hopea racophloea* | *Monotes africanus* |
| *Hopea recopei* | *Monotes autennei* |
| *Hopea reticulata* | *Monotes dasyanthus* |
| *Hopea rudiformis* | *Monotes doryphorus* |
| *Hopea rugifolia* | *Monotes duvigneaudii* |
| *Hopea samarensis* | *Monotes engleri* |
| *Hopea sangal* | *Monotes glaber* |
| *Hopea scabra* | *Monotes glandulosus* |
| *Hopea semicuneata* | *Monotes gossweileri* |
| *Hopea shingkeng* | *Monotes hirtii* |
| *Hopea similis* | *Monotes hypoleucus* |
| *Hopea sphaerocarpa* | *Monotes katangensis* |
| *Hopea subalata* | *Monotes kerstingii* |
| *Hopea sublanceolata* | *Monotes lutambensis* |
| *Hopea sulcata* | *Monotes madagascariensis* |
| *Hopea tenuinervula* | *Monotes magnificus* |
| *Hopea thorelii* | *Monotes paivae* |
| *Monotes pearsonii* | *Shorea almon* |
| *Monotes redheadii* | *Shorea altopoensis* |
| *Monotes rubriglans* | *Shorea alutacea* |
| *Monotes rufotomentosus* | *Shorea amplexicaulis* |
| *Monotes xasenguensis* | *Shorea andulensis* |
| *Neobalanocarpus heimii* | *Shorea angustifolia* |
| *Pakaraimaea dipterocarpacea* | *Shorea argentea* |
| *Parashorea aptera* | *Shorea argentifolia* |
| *Parashorea buchananii* | *Shorea asahii* |
| *Parashorea chinensis* | *Shorea assamica* |
| *Parashorea densiflora* | *Shorea astylosa* |
| *Parashorea dussaudii* | *Shorea atrinervosa* |
| *Parashorea globosa* | *Shorea bakoensis* |
| *Parashorea lucida* | *Shorea balangeran* |
| *Parashorea macrophylla* | *Shorea balanocarpoides* |
| *Parashorea malaanonan* | *Shorea beccariana* |
| *Parashorea parvifolia* | *Shorea bentongensis* |
| *Parashorea smythiesii* | *Shorea biawak* |
| *Parashorea stellata* | *Shorea blumutensis* |
| *Parashorea tomentella* | *Shorea bracteolata* |
| *Parashorea warburgii* | *Shorea brunnescens* |
| *Pseudomonotes tropenbosii* | *Shorea bullata* |
| *Shorea acuminata* | *Shorea calcicola* |
| *Shorea acuminatissima* | *Shorea cara* |
| *Shorea acuta* | *Shorea carapae* |
| *Shorea affinis* | *Shorea chaiana* |
| *Shorea agamii* | *Shorea ciliata* |
| *Shorea albida* | *Shorea collaris* |
| *Shorea collina* | *Shorea flemmichii* |
| *Shorea confusa* | *Shorea foraminifera* |
| *Shorea congestiflora* | *Shorea foxworthyi* |
| *Shorea conica* | *Shorea furfuracea* |
| *Shorea contorta* | *Shorea gardneri* |
| *Shorea cordata* | *Shorea geniculata* |
| *Shorea cordifolia* | *Shorea gibbosa* |
| *Shorea coriacea* | *Shorea glauca* |
| *Shorea crassa* | *Shorea gratissima* |
| *Shorea curtisii* | *Shorea guiso* |
| *Shorea cuspidata* | *Shorea havilandii* |
| *Shorea dasyphylla* | *Shorea hemsleyana* |
| *Shorea dealbata* | *Shorea henryana* |
| *Shorea dispar* | *Shorea hopeifolia* |
| *Shorea disticha* | *Shorea hulanidda* |
| *Shorea domatiosa* | *Shorea hypochra* |
| *Shorea dyeri* | *Shorea hypoleuca* |
| *Shorea elliptica* | *Shorea iliasii* |
| *Shorea exelliptica* | *Shorea inaequilateralis* |
| *Shorea faguetiana* | *Shorea inappendiculata* |
| *Shorea faguetioides* | *Shorea induplicata* |
| *Shorea falcata* | *Shorea isoptera* |
| *Shorea falcifera* | *Shorea javanica* |
| *Shorea falciferoides* | *Shorea johorensis* |
| *Shorea fallax* | *Shorea kuantanensis* |
| *Shorea farinosa* | *Shorea kudatensis* |
| *Shorea ferruginea* | *Shorea kunstleri* |
| *Shorea flaviflora* | *Shorea ladiana* |
| *Shorea laevis* | *Shorea oblongifolia* |
| *Shorea lamellata* | *Shorea obovoidea* |
| *Shorea laxa* | *Shorea obscura* |
| *Shorea lepidota* | *Shorea obtusa* |
| *Shorea leprosula* | *Shorea ochracea* |
| *Shorea leptoderma* | *Shorea ochrophloia* |
| *Shorea lissophylla* | *Shorea ovalifolia* |
| *Shorea longiflora* | *Shorea ovalis* |
| *Shorea longisperma* | *Shorea ovata* |
| *Shorea lumutensis* | *Shorea pachyphylla* |
| *Shorea lunduensis* | *Shorea palembanica* |
| *Shorea macrantha* | *Shorea pallescens* |
| *Shorea macrobalanos* | *Shorea pallidifolia* |
| *Shorea macrophylla* | *Shorea palosapis* |
| *Shorea macroptera* | *Shorea parvifolia* |
| *Shorea malibato* | *Shorea parvistipulata* |
| *Shorea materialis* | *Shorea patoiensis* |
| *Shorea maxima* | *Shorea pauciflora* |
| *Shorea maxwelliana* | *Shorea peltata* |
| *Shorea mecistopteryx* | *Shorea pilosa* |
| *Shorea megistophylla* | *Shorea pinanga* |
| *Shorea micans* | *Shorea platycarpa* |
| *Shorea monticola* | *Shorea platyclados* |
| *Shorea montigena* | *Shorea polita* |
| *Shorea mujongensis* | *Shorea polyandra* |
| *Shorea multiflora* | *Shorea polysperma* |
| *Shorea myrionerva* | *Shorea praestans* |
| *Shorea negrosensis* | *Shorea pubistyla* |
| *Shorea quadrinervis* | *Shorea submontana* |
| *Shorea resinosa* | *Shorea sumatrana* |
| *Shorea retinodes* | *Shorea superba* |
| *Shorea retusa* | *Shorea symingtonii* |
| *Shorea revoluta* | *Shorea tenuiramulosa* |
| *Shorea richetia* | *Shorea teysmanniana* |
| *Shorea robusta* | *Shorea thorelii* |
| *Shorea rogersiana* | *Shorea trapezifolia* |
| *Shorea rotundifolia* | *Shorea tumbuggaia* |
| *Shorea roxburghii* | *Shorea uliginosa* |
| *Shorea rubella* | *Shorea venulosa* |
| *Shorea rubra* | *Shorea virescens* |
| *Shorea rugosa* | *Shorea waltoni* |
| *Shorea sagittata* | *Shorea wangtianshuea* |
| *Shorea scaberrima* | *Shorea woodii* |
| *Shorea scabrida* | *Shorea worthingtoni* |
| *Shorea scrobiculata* | *Shorea xanthophylla* |
| *Shorea selanica* | *Shorea zeylanica* |
| *Shorea seminis* | *Stemonoporus acuminatus* |
| *Shorea siamensis* | *Stemonoporus affinis* |
| *Shorea singkawang* | *Stemonoporus angustisepalus* |
| *Shorea slootenii* | *Stemonoporus bullatus* |
| *Shorea smithiana* | *Stemonoporus canaliculatus* |
| *Shorea splendida* | *Stemonoporus cordifolius* |
| *Shorea squamata* | *Stemonoporus elegans* |
| *Shorea stenoptera* | *Stemonoporus gardneri* |
| *Shorea stipularis* | *Stemonoporus gilimalensis* |
| *Shorea subcylindrica* | *Stemonoporus gracilis* |
| *Stemonoporus kanneliyensis* | *Vatica borneensis* |
| *Stemonoporus laevifolius* | *Vatica brevipes* |
| *Stemonoporus lanceolatus* | *Vatica brunigii* |
| *Stemonoporus lancifolius* | *Vatica cauliflora* |
| *Stemonoporus latisepalus* | *Vatica chartacea* |
| *Stemonoporus marginalis* | *Vatica chevalieri* |
| *Stemonoporus mooni* | *Vatica chinensis* |
| *Stemonoporus nitidus* | *Vatica cinerea* |
| *Stemonoporus oblongifolius* | *Vatica compressa* |
| *Stemonoporus petiolaris* | *Vatica congesta* |
| *Stemonoporus reticulatus* | *Vatica coriacea* |
| *Stemonoporus revolutus* | *Vatica cuspidata* |
| *Stemonoporus rigidus* | *Vatica diospyroides* |
| *Stemonoporus scalarinervis* | *Vatica dulitensis* |
| *Stemonoporus scaphifolius* | *Vatica elliptica* |
| *Stemonoporus zeylanicus* | *Vatica endertii* |
| *Upuna borneensis* | *Vatica flavida* |
| *Vateria copallifera* | *Vatica flavovirens* |
| *Vateria indica* | *Vatica glabrata* |
| *Vateria macrocarpa* | *Vatica globosa* |
| *Vateriopsis seychellarum* | *Vatica granulata* |
| *Vatica abdulrahmaniana* | *Vatica griffithii* |
| *Vatica adenanii* | *Vatica guangxiensis* |
| *Vatica affinis* | *Vatica harmandiana* |
| *Vatica albiramis* | *Vatica havilandii* |
| *Vatica badiifolia* | *Vatica heteroptera* |
| *Vatica bantamensis* | *Vatica hullettii* |
| *Vatica bella* | *Vatica javanica* |
| *Vatica lanceifolia* | *Vatica sarawakensis* |
| *Vatica lobata* | *Vatica scortechinii* |
| *Vatica lowii* | *Vatica soepadmoi* |
| *Vatica maingayi* | *Vatica stapfiana* |
| *Vatica mangachapoi* | *Vatica subglabra* |
| *Vatica maritima* | *Vatica teysmanniana* |
| *Vatica micrantha* | *Vatica thorelii* |
| *Vatica mizaniana* | *Vatica umbonata* |
| *Vatica nitens* | *Vatica venulosa* |
| *Vatica oblongifolia* | *Vatica vinosa* |
| *Vatica obovata* | *Vatica xishuangbannaensis* |
| *Vatica obscura* | *Vatica yeechongii* |
| *Vatica odorata* |  |
| *Vatica pachyphylla* |  |
| *Vatica pallida* |  |
| *Vatica paludosa* |  |
| *Vatica palungensis* |  |
| *Vatica parvifolia* |  |
| *Vatica patentinervia* |  |
| *Vatica pauciflora* |  |
| *Vatica pedicellata* |  |
| *Vatica pentandra* |  |
| *Vatica perakensis* |  |
| *Vatica philastreana* |  |
| *Vatica rassak* |  |
| *Vatica ridleyana* |  |
| *Vatica rotata* |  |
| *Vatica rynchocarpa* |  |

**Appendix C2**: Dipterocarp phylogeny in three scenarios. A) Scenario One; B) Scenario Two; C) Scenario Three


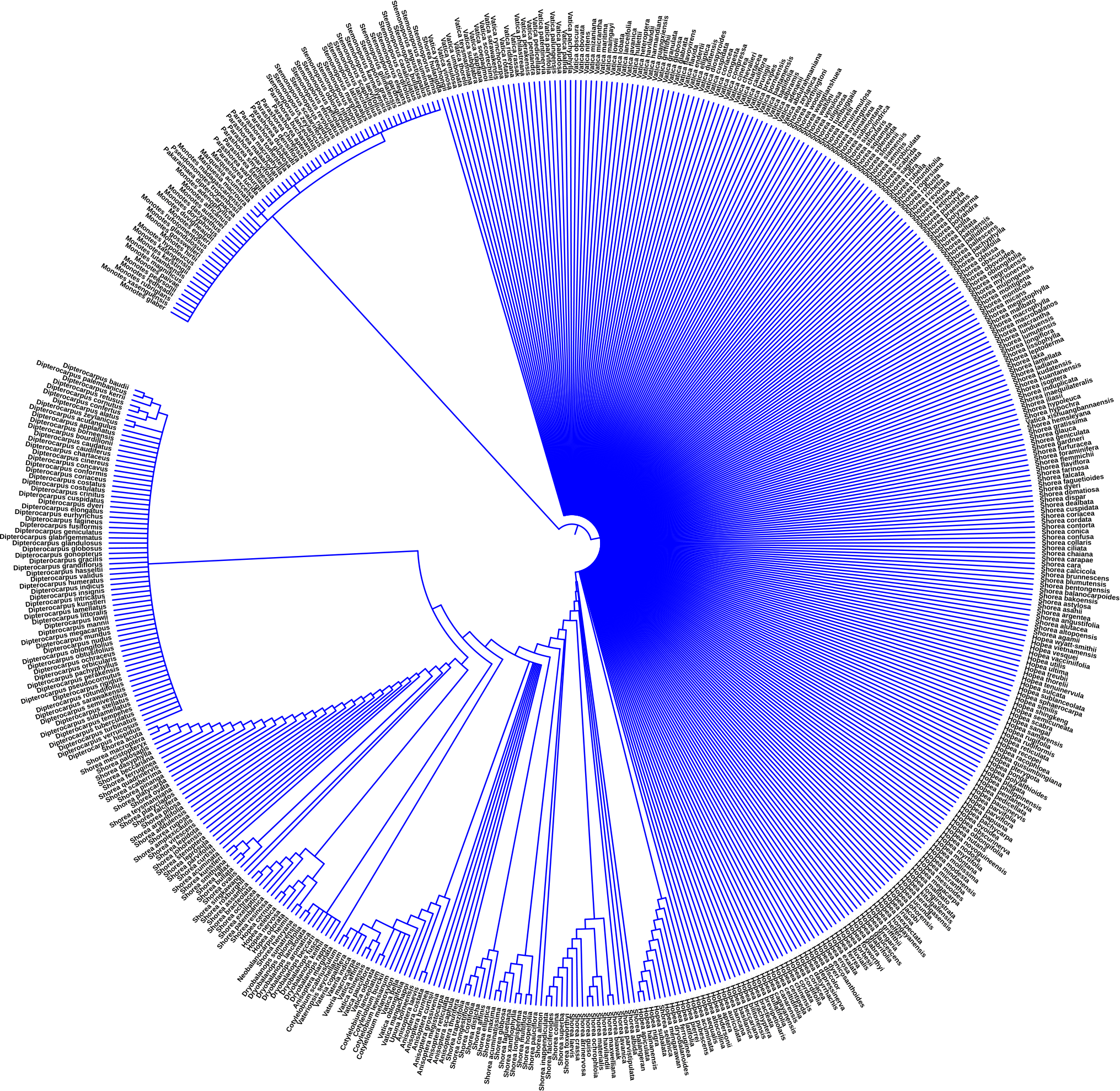


**A) Scenario One**


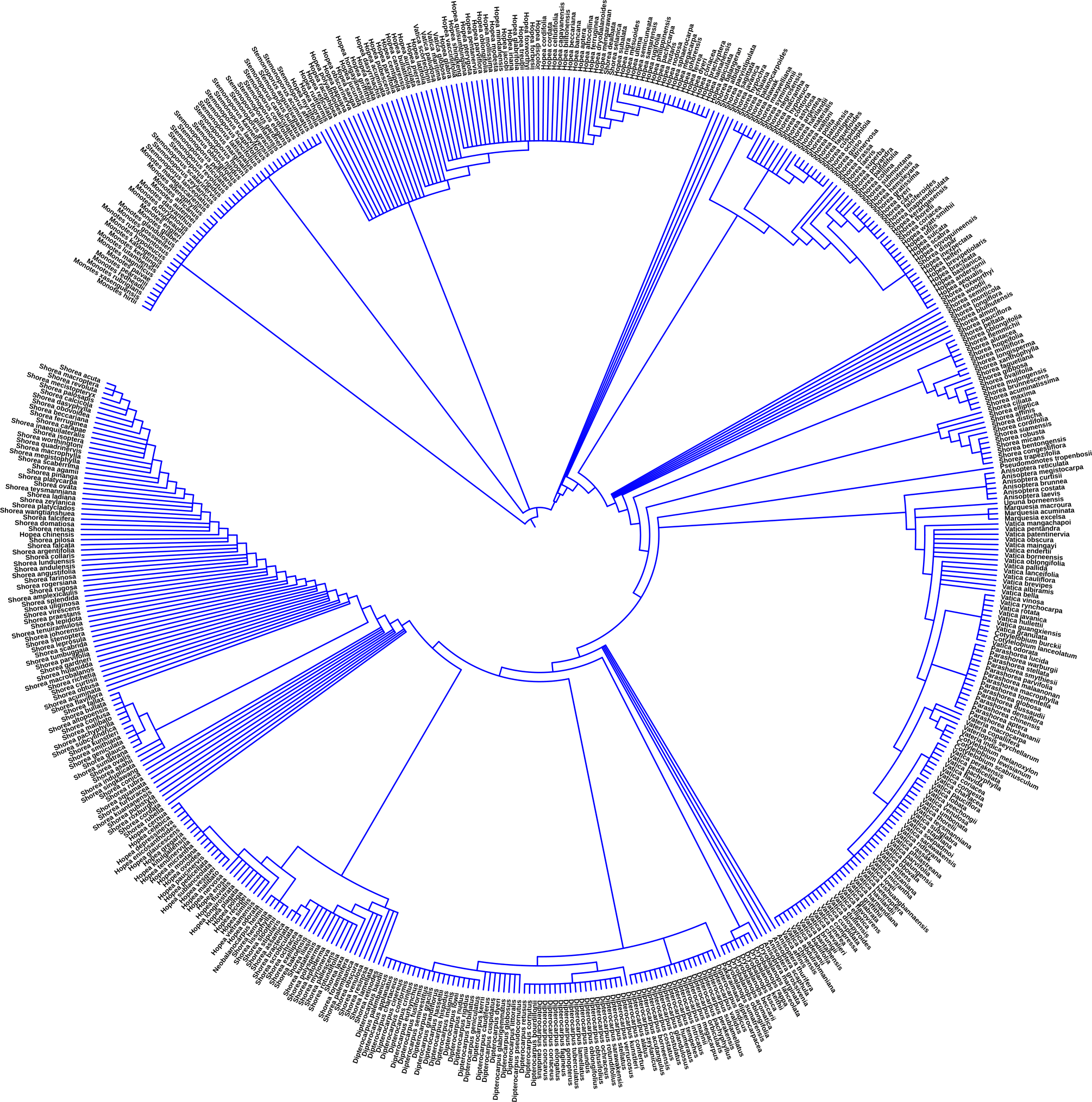


**B) Scenario Two**


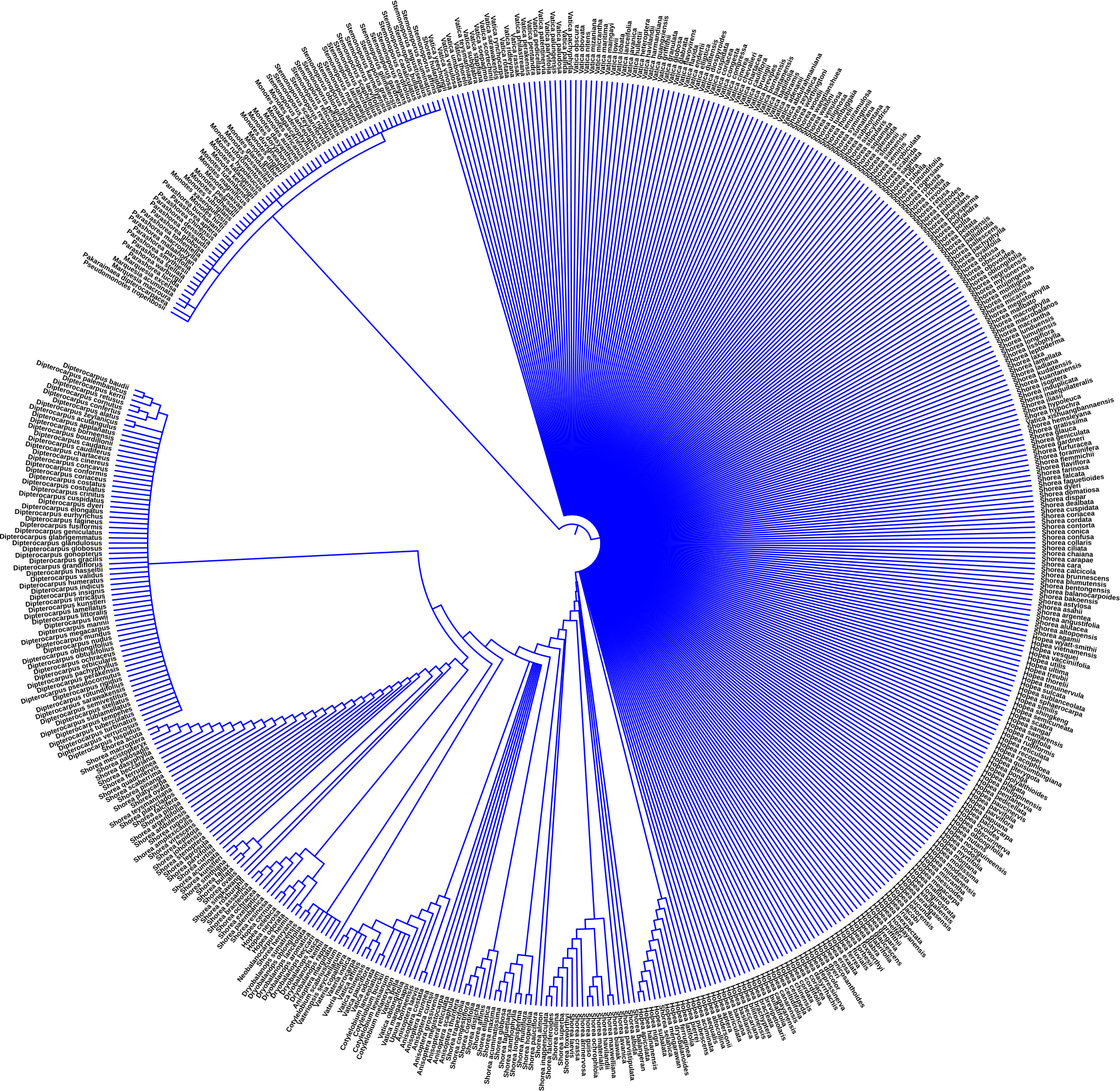


**C) Scenario Three**
